# Two Visual Pathways in Primates Based on Sampling of Space: Exploitation and Exploration of Visual Information

**DOI:** 10.3389/fnint.2016.00037

**Published:** 2016-11-22

**Authors:** Bhavin R. Sheth, Ryan Young

**Affiliations:** ^1^Department of Electrical and Computer Engineering, University of HoustonHouston, TX, USA; ^2^Center for NeuroEngineering and Cognitive Systems, University of HoustonHouston, TX, USA; ^3^Department of Neuroscience, Brandeis UniversityWaltham, MA, USA

**Keywords:** fovea, periphery, embodied cognition, bayes, dorsal stream, ventral stream, visual cortex, cortical organization

## Abstract

Evidence is strong that the visual pathway is segregated into two distinct streams—ventral and dorsal. Two proposals theorize that the pathways are segregated in function: The ventral stream processes information about object identity, whereas the dorsal stream, according to one model, processes information about either object location, and according to another, is responsible in executing movements under visual control. The models are influential; however recent experimental evidence challenges them, e.g., the ventral stream is not solely responsible for object recognition; conversely, its function is not strictly limited to object vision; the dorsal stream is not responsible by itself for spatial vision or visuomotor control; conversely, its function extends beyond vision or visuomotor control. In their place, we suggest a robust dichotomy consisting of a ventral stream selectively sampling high-resolution/*focal* spaces, and a dorsal stream sampling nearly all of space with reduced foveal bias. The proposal hews closely to the theme of embodied cognition: Function arises as a consequence of an extant sensory underpinning. A continuous, not sharp, segregation based on function emerges, and carries with it an undercurrent of an exploitation-exploration dichotomy. Under this interpretation, cells of the ventral stream, which individually have more punctate receptive fields that generally include the fovea or parafovea, provide detailed information about object shapes and features and lead to the systematic exploitation of said information; cells of the dorsal stream, which individually have large receptive fields, contribute to visuospatial perception, provide information about the presence/absence of salient objects and their locations for novel exploration and subsequent exploitation by the ventral stream or, under certain conditions, the dorsal stream. We leverage the dichotomy to unify neuropsychological cases under a common umbrella, account for the increased prevalence of multisensory integration in the dorsal stream under a Bayesian framework, predict conditions under which object recognition utilizes the ventral or dorsal stream, and explain why cells of the dorsal stream drive sensorimotor control and motion processing and have poorer feature selectivity. Finally, the model speculates on a dynamic interaction between the two streams that underscores a unified, seamless perception. Existing theories are subsumed under our proposal.

Brain mass and volume are fundamental constraints in the development of brains in general and in the development of the primate brain in particular. Primate brains are large. Brain mass of primates, humans as well as non-humans, is substantial, both in absolute terms and in proportion to body mass (Striedter, 2005). Experimental measurements suggest that large brains are associated with sparse connectivity among brain areas: While the inter-areal cortical connection weight distribution is well fit by a lognormal distribution in both primate and mouse, the range of weight values is significantly narrower (10^2^-fold) in mouse compared to monkey (10^5^-fold) (Wang and Kennedy, 2016). This is in accord with the idea that in the mouse, brain areas connect to one another whereas in the macaque, the inter-areal connections are more selective and sparse. Theoretical arguments as well support the view that brain size limits the strength of inter-areal connectivity (Striedter, 2005). Consider a brain consisting of N areas: In order for all the areas to directly communicate with one another, O(N^2^) connections will be required. While species with smaller brains can and do afford the luxury of all brain areas projecting directly to one another, the requirement of pairwise connectivity in primate brains could lead to an explosion of white matter making it all but impossible in reality to have all the brain areas directly talk with one another. Therefore, there must be specificity in brain wiring, so that certain areas directly talk to a subset, but not all. As a result, there is considerable pressure for there to be segregation of brain pathways in the primate brain (Striedter, 2005).

The visual modality is arguably the most developed in the primate and occupies the largest amount of real estate: approximately 50% of cerebral cortex in macaque and 20–30% in humans is devoted to visual processing (Van Essen, 2004). Thus, on the basis of the above discussion about the need for segregation of large brains, the segregation of the visual brain of primates into pathways, in which areas belonging to a given pathway talk directly with each other more than with areas belonging to a different pathway, is likely to be seen. Recent studies by Kennedy et al. (Markov et al., 2013; Wang and Kennedy, 2016) have revealed important information about cortical connectivity between different cortical areas, or inter-areal cortical connection, which is consistent with the idea of (at least) two somewhat segregated visual pathways. Whereas structural connections exist between most areas (66% of the cortical area interconnectivity matrix is nonzero), of greater importance is that the strength of inter-areal connections is not uniform and there is segregation of connectivity that is consistent with the idea of two visual pathways (see Figure 1B and Figure 3 of Markov et al., 2013). It bears mention here that the studies measure structural connectivity—and not functional connectivity—which tends to be less selective and coarser. Furthermore, the strength of connectivity decays exponentially with inter-areal distance so that nearby areas connect to one another more strongly than areas farther away, which too is in line with the idea that the two pathways are segregated to some extent, i.e., the nearby areas of the ventral stream preferentially connect to one another while those of the dorsal stream preferentially connect to one another.

Apropos, there has been not one, but two influential models for the segregation of visual information in the primate brain—one based on studies conducted in non-human primates (*macaca mulata, macaca nemestrima, macaca fascicularis, and macaca fuscata*) and another based on neurological case studies in human. Ungerleider and Mishkin (1982) proposed an influential model for the segregation of visual pathways into two streams based on anatomical location—a dorsal stream and a ventral stream (Figure 1). The “what/where” model was based largely on lesion studies of non-human primates in which they proposed that the ventral stream is responsible for object vision and the dorsal stream for spatial vision. According to them, lesions of the ventral stream affects monkeys' ability either to recognize objects, whereas lesions of the dorsal stream affect monkey's ability to locate an object in space relative to a reference. Goodale et al. observing human case studies—in particular, the celebrated patient DF (Goodale et al., 1991), proposed a modification of the previous model (Goodale and Milner, 1992). In the new “perception/action” model, the function of the ventral stream was the same as before, but that of the dorsal stream was changed. From their model, the ventral stream processes visual information for the purpose of visual perception (“vision for perception”), while the dorsal stream processes visual information for the purpose of executing movements (“vision for action”). The models have existed side by side for over 20 years and each has been influential in shaping the thinking of generations of vision researchers and neuroscientists. It should be noted that the two models interpret experimental data differently. Some share the belief that the “spatial localization” and “vision for action” models of the dorsal stream go hand in hand, as central to the “vision for action” model is the need to localize a target in order to fixate with the eye or grasp with the hand. However, as stated in Pisella et al. (2013a), neuroimaging data has revealed that the involvement of the dorsal stream is not restricted within the context of motor actions (Faillenot et al., 1999; Konen and Kastner, 2008) and, besides deficits in visuomotor control, lesions of the dorsal stream may have devastating consequences on the global perception of a visual scene (Balint, 1909; Pisella et al., 2008, 2013b), on visuospatial perception (Pisella et al., 2013a), and on *sensori*motor control in general, e.g., proprioceptive localization of body parts in space (Blangero et al., 2007). Thus, the two models are not one and the same and there are results that are consistent with one but not with the other.

**Figure 1:**
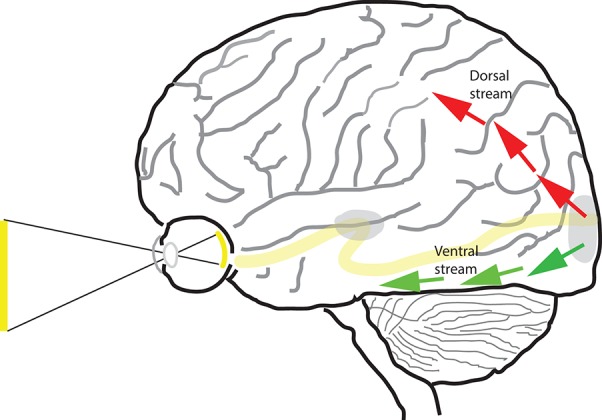
**The dorsal and ventral streams of the visual pathway**. Beyond area V1 (shown at occipital pole) and V2 of the cortex, the visual pathway is segregated into two separate pathways—dorsal (red arrows) and ventral (green arrows).

Reconciliation between the two leading models is *a* goal of the present article. We explain briefly the salient points of each model, then point out a few of the key limitations and problems with each, and offer our model as a step toward reconciliation. The model provides a general unifying scaffold for both models to rest on, but is broader and more comprehensive than each. Finally, we discuss the benefits of the proposed model and its implications.

## Limitations of what/where dichotomy

The what/where model, i.e., the ventral stream processes object vision, the dorsal stream processes spatial vision, has been enormously influential. However, since its publication 30 years ago, experimental evidence has accumulated challenging the model. The arguments against the model fall along two broad lines: first, the ventral stream is not solely responsible for object recognition; conversely, the ventral stream does more than just participate in object vision. Second, the dorsal stream's role is not confined to spatial vision but rather is more general; the dorsal stream contributes to visual attention and more generally, to *spatial* attention. The critical roles of the dorsal stream in spatial attention and in sensorimotor control are neither directly explained nor indirectly implied by the what/where model. We discuss a sample of the arguments for each of these points below.

First, although the ventral visual pathway is primarily responsible for the processing of spatial details and high resolution visual features (and features like color which are processed mainly in the visual center), it is not the case that it alone is responsible for the recognition of objects. The dorsal stream also participates in object recognition, at least under certain conditions, i.e., when the stimuli are novel, unconventional or challenging in some way, or when the integration of features across an expanse of space, or across several fixations, is required. Object selective responses have been found in cortical areas IPS1 and IPS2 of the dorsal stream to objects with semantic content (2D line drawings of common objects and tools) and without (2D and 3D objects like stars and spheres; Konen and Kastner, 2008). Cortical areas belonging to the dorsal stream have been found to be responsive to stimulus features supposed to be processed in the ventral stream e.g., shape selectivity has been found in neurons of lateral intraparietal area (LIP) of monkey cortex (Sereno and Maunsell, 1998), transformation-invariant object-selective responses in areas IPS1 and IPS2 of human cortex (Konen and Kastner, 2008), color selective response in monkey area LIP when color cued the direction of eye movement (Toth and Assad, 2002), and color discrimination in human dorsal IPS (Claeys et al., 2004). fMRI studies in the anesthetized monkey have revealed 3D shape-specific activation in a number of areas of the dorsal pathway including V3A, LIP, LOP and the FEF (Shikata et al., 1996; Sereno et al., 2002; Durand et al., 2007, 2009); because the shape-specific activation was observed in an anesthetized monkey, it reflects automatic processing of 3D shape divorced from the influences of attention, intention, or memory^1^. Patient AF has a lesion that involves dorsally the occipital-parietal area, including the region of the temporal-parietal-occipital junction and has an otherwise intact ventral stream (Vaina, 1994); however, he is impaired at recognizing objects presented from unconventional views, although recognition of prototypical views of objects, and color and form discrimination are normal, as is his ability to recognize faces. Apropos, based on neurological case studies, it has been claimed that perceptual categorization and the recognition of objects viewed from an unfamiliar perspective is affected in cases of right parietal lesions (Warrington and Taylor, 1973, 1978), and parietal lesions impair the perception of pictures in humans, especially if the pictures are, in some way, incomplete (Ettlinger, 1960; De Renzi and Spinnler, 1966; Warrington and James, 1967). Last but not least, dorsal simultanagnosia is a condition in which patients have bilateral parieto-occipital lesions affecting the dorsal pathway; the patients can recognize objects but cannot see more than one object at a time (except on rare occasions when the multiple objects are small, close together, and foveal), and attentional impairment is influenced strongly by the boundaries of objects (Luria, 1959; Humphreys and Riddoch, 1993), again indicating a role for the dorsal pathway in certain forms of object recognition, especially for the integration and figure-ground segmentation of separable features across space, which requires larger receptive fields (that typically include the visual periphery).

Conversely, Ettlinger (Ettlinger, 1990) has argued that object vision is too narrow a function for the ventral stream: responses to complex objects are observed only in areas TE and TEO (Desimone et al., 1984) of the ventral visual pathway, and responses to complex conjunctions of features is confined to the perirhinal cortex (Buckley and Gaffan, 2006; Lehky and Tanaka, 2016). Thus, object vision does not account for the function of other areas of the ventral stream, e.g., V4, V3v. Rather, the ventral stream is likely to be contributing toward the discrimination of a number of visual features such as size, shape, color, brightness; therefore, the ventral stream is likely to play a crucial, albeit not exclusive, role in *feature* vision, not object vision. In fact, no real evidence exists arguing for the role of the ventral stream in the recognition of objects in isolation from a putative role in the discrimination of visual features. Clearly, a complex conjunction of features is no different philosophically from an “object,” and eventually object recognition is a function of the ventral visual pathway. However, as argued above, this is an incomplete description: allowance has to be made for the extraction of information about features (e.g., color, texture, shape etc.) of an item, or features of even multiple items within a focal area of interest, without concomitant object identification. The above findings and arguments, and others, weaken the argument for the strong version of the object vision/spatial vision dichotomy.

Second, the dorsal stream plays an equally, if not more, critical role in spatial attention as it does in spatial vision. Ungerleider and Mishkin (1982) themselves cited “…contralateral neglect of auditory and tactile as well as visual stimuli…” following parietal lesions in monkey, which is not an argument for visual attention but rather for the more inclusive category of spatial attention (note later reports of extinction, not neglect, following parietal lesions in the monkey (Lynch and McLaren, 1989), and neglect following frontal lesions in the monkey instead (e.g., Deuel and Collins, 1983; Rizzolatti et al., 1983). Others have argued that visuospatial perception is not the only function affected following lesions of the parietal cortex in the monkey (Mendoza and Thomas, 1975; Lawler and Cowey, 1987): Following unilateral parietal lesions in the monkey, goal-directed reaching with the contralesional hand is usually more inaccurate than that the ipsilesional one (implying the monkey knows *where* the target is but cannot reach it with its contralesional hand; this is termed the hand effect in the literature and is an additional component to the visuospatial deficit), which means the deficit is not a purely visual one. An individual with a parietal lesion fares worse in the dark than in the light (implying a disorder of spatial orientation), which argues, again, that the deficit is not purely visual. Furthermore, patients with dorsal simultanagnosia arising from bilateral damage to the parieto-occipital cortex show deficits in both spatial vision and spatial attention. Patients fail to point to or reach for a recognized visual stimulus or to describe its location, which argues for a role of the dorsal pathway in spatial vision; this inability to localize stimuli even when they are seen is called visual disorientation. At the same time, they show remarkable deficits in spatial attention: they cannot see more than one stimulus at a time and have a hard time shifting their gaze and focus from one stimulus to another. These show up as apparent deficits in motor control as they grope for things in the dark and walk into furniture and so on, and yet their deficits have an underlying cause rooted in attention. Farah (2004) has argued that deficits in spatial localization are secondary to and perhaps caused by deficits in spatial attention, arguing for the primacy of spatial attention. This is because, she argues, the location of an object can be specified only relative to another location, be it the subject's own body (in a pointing or reaching task), another object (when describing the object's location relative to another), or the origin of some abstract coordinate system. From this logic, the inability of dorsal simultanagnosics to attend to two separate loci would therefore be expected to impair localization. Moreover, tests of spatial localization (of dots on frames) on patients with optic ataxia, which arises from damage to the dorsal pathway, found that when spatial attention was brought to bear vs. when it was not, performance of said patients was impaired to a greater extent (T6 - T5 difference scores in Pisella et al., 2013a). Thus, the study revealed attentional dysfunction in ataxia that went above and beyond visuo-spatial integration deficits. Findings such as these lead one to conclude that the function of the dorsal stream is not merely spatial vision; rather, it is involved in visual, and more generally, spatial attention, and in the processing of modalities other than vision as well as in spatial memory (and not just on-line spatial vision).

In brief, there are several issues with the what/where model's interpretation of the dorsal and ventral streams. The ventral stream is likely not contributing toward object vision alone but is involved in the processing of a number of visual features; the ventral stream is not solely responsible for object recognition; the dorsal stream is responsible for spatial vision as well as for spatial attention, and is responsible for object recognition under certain conditions.

## Limitations of perception action model

The perception action model, like the what/where model, states that the ventral stream is responsible for object vision/perception. Some of the issues with this interpretation, i.e., the perception part of the model, were discussed above.

There are issues with the action part of the perception action model as well. Neurological case studies—patients with optic ataxia—argue for a more limited role of the dorsal stream in visuomotor control. Optic ataxia is a neurological disorder that arises from damage to certain parts of the parietal lobe, and is sometimes acclaimed as the dorsal stream counterpart to ventral visual form agnosia (patient DF); behavioral symptoms arising in patients with optic ataxia are considered to be classic deficits in vision for action. However, this interpretation is problematic. Almost all optic ataxia studies emphasize that central foveal vision is relatively unimpaired in patients with optic ataxia (Perenin and Vighetto, 1988; Milner et al., 1999; Pisella et al., 2000; Battaglia-Mayer and Caminiti, 2002; Rossetti et al., 2003), and that peripheral vision and perception (Rossetti et al., 2005a,b), are impaired instead; the frequency of reaching errors and error magnitudes increase with retinal eccentricity of target location. In other words, action is unimpaired in patients with dorsal stream damage if it involves central parts of the visual field. In sum, the neurological case studies further highlight the restricted role of the dorsal stream in vision for action. On a related note, Balan and Gottlieb (2009) showed that the inactivation of LIP, an area of the dorsal stream, did not cause global or limb-specific deficits in manual release.

Apropos, in addition to on-line peripheral vision being dependent on the dorsal stream, studies have found that spatial working memory is affected specifically in neglect patients with lesions including the posterior parietal cortex or PPC (Pisella and Mattingley, 2004), suggesting a role for parts of the dorsal stream in functions that have little in common with on-line motor action. A classic deficit in these patients is an “amnesic aspect of exploration (Pisella et al., 2006, p. 2745),” called revisiting behavior, the inability to remember previously scanned locations and the tendency to revisit them. It has been postulated that re-visiting behavior could be accounted for by a disorder of spatial trans-saccadic remapping processes (Pisella and Mattingley, 2004), which have been shown to operate in higher-level oculocentric maps of the PPC to ensure visual integration of the successive retinal images over time and space (Heide et al., 1995).

Recent work on DF, the celebrated patient who has extensive ventral stream damage adds fuel to the above argument. Pointing and grasping of objects placed in the periphery under open- and closed-loop conditions is significantly worse in patient DF (Hesse et al., 2014), although pointing and grasping is relatively intact for objects placed near the fovea under similar open- and closed-loop conditions. Early, high resolution MRI studies have revealed a caveat, however: DF's brain damage includes the ventral stream as well as a unilateral lesion to her left posterior parietal cortex of the dorsal stream (James et al., 2003) and significantly reduced cortical thickness in the posterior intraparietal sulcus (IPS) of both hemispheres (Bridge et al., 2013)—a cortical area frequently implicated in optic ataxia. These claims have not been confirmed yet, but if verified, they would suggest that DF is no longer an example of pure ventral stream damage (Hesse et al., 2014). More generally, the evidence supports the argument that neither the ventral nor the dorsal stream is critical for all aspects of visually guided goal-directed behavior. Instead, a more parsimonious account of the pointing behavior of patients with optic ataxia (see Rossetti et al., 2003), is that the *critical and exclusive* visuomotor function of the dorsal stream is restricted to the processing of visual targets presented in the periphery in the fixation condition, i.e., where the individual points to targets presented in the periphery while maintaining fixation at a fixed location (as opposed to the more naturalistic free viewing condition, in which the individual can move their eyes freely including to the target presented). (Note that we do not interpret the above results as suggesting that the dorsal stream is not involved in action preparation toward the center of the visual field—it is involved—, but rather that there is redundant coding of the center in the visual brain; therefore, loss of the dorsal stream has relatively less impact on action preparation toward the center. Indeed, the ventral visual pathway has a strong representation of the center of the visual field).

Conversely, neurological case studies suggest that the dorsal stream does more than just visuomotor control. Michel and Henaff (2004), when discussing the case of a patient AT with dorsal simultanagnosia, state: “AT's deficit cannot be reduced to a visuo-motor deficit. The present work emphasizes a relatively neglected deficit in such patients, that is, a visual attentional deficit. This deficit comprises both an inability to see two items at the same time and a striking difficulty to shift the locus of foveation. Thus, when one specifies the dorsal system as being a visuo-motor system, one must not neglect the word “visuo” which points to the perceptual capacities of the parietal lobe.”: The argument that the dorsal stream plays a critical role in spatial attention, and not spatial vision or visuomotor control, appears to be a recurring theme in studies arguing against the two models. In summary, these and other arguments suggest that the role of the dorsal stream in visuomotor control, i.e., vision for action, is not as incontrovertible as it appears and that the dorsal stream is likely to be involved in functions beyond visuomotor control, namely in spatial attention.

Other studies have revealed other functions for the dorsal pathway that go beyond visuomotor control. Studies have found that spatial working memory is affected specifically in neglect patients with lesions including the posterior parietal cortex (Pisella and Mattingley, 2004), suggesting a role for parts of the dorsal stream in functions that have little in common with on-line motor action. A classic deficit in these patients is an “amnesic aspect of exploration (Pisella et al., 2006, p. 2745),” called revisiting behavior, the inability to remember previously scanned locations and the tendency to revisit them. It has been postulated that re-visiting behavior could be accounted by a disorder of spatial trans-saccadic remapping processes (Pisella and Mattingley, 2004), which has been shown to operate in higher-level oculocentric maps of the parietal cortex to ensure visual integration of the successive retinal images over time and space (Heide et al., 1995). Along similar lines as above, a recent study showed that reward associations modify not only the representation of an upcoming saccade but also the bottom-up salience of a visual stimulus independently of a motor output (Peck et al., 2009). The finding is suggestive of a function for LIP (and the dorsal stream in general) that is more general than simply visuomotor control.

## Our proposal: exploration and exploitation

The what/where and perception action models have been influential in shaping neuroscience research. One might argue that researchers have reinterpreted their experimental findings to accommodate them to said models. Limitations and drawbacks of the two influential approaches, each of which was proposed over two decades ago, have led us to develop a new proposal for the segregation of visual information into two separate pathways.

The proposal is not based on the primacy of function, but rather on the primacy of connections and receptive field structure. In our proposal, function is an emergent property arising from a combination of factors including the heterogeneity of retinal input, pattern of connections between subcortical structures and the cortex and corticocortical connections, neural responses and differences inherent therein.

**Figure 2:**
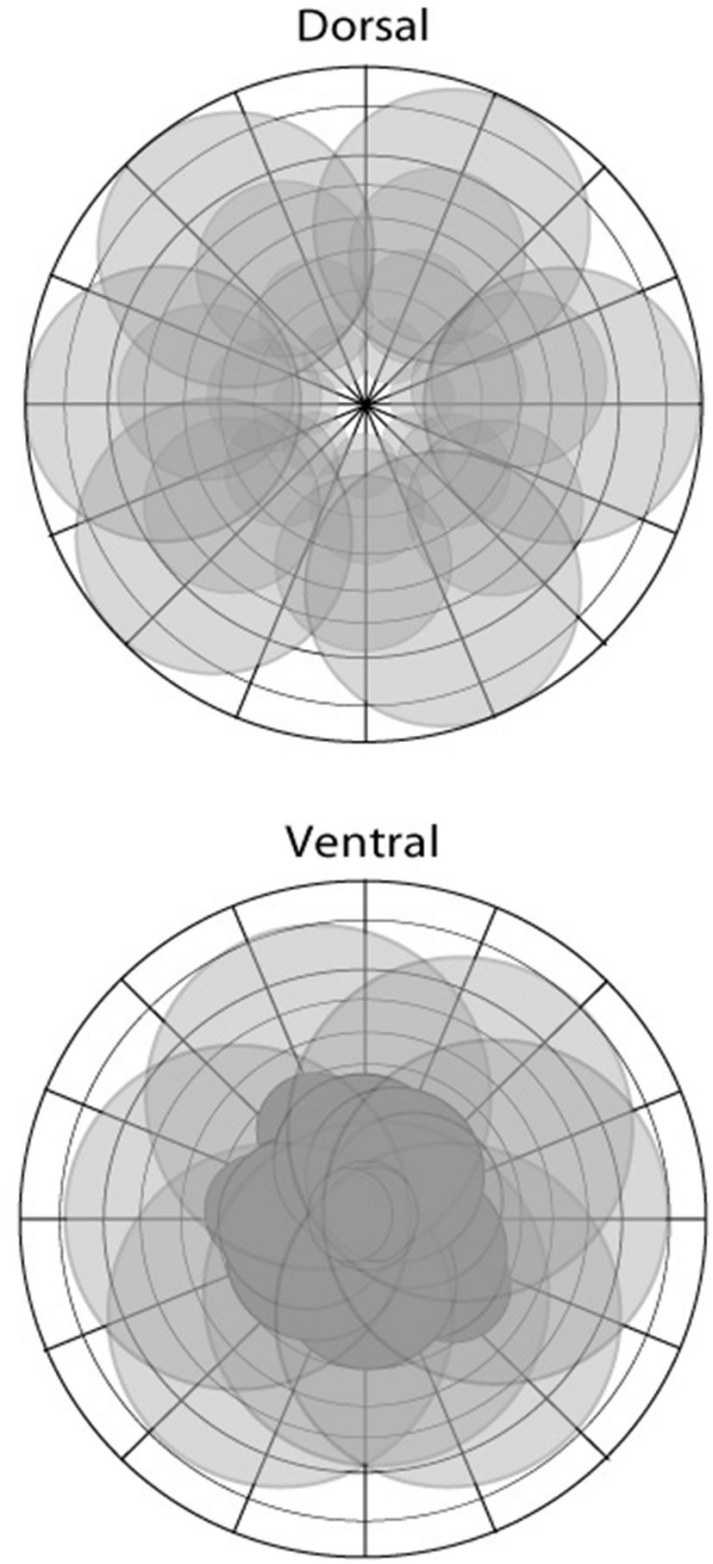
**The representation of visual space in areas of the dorsal (top) and ventral (bottom) pathways illustrated in schematic form**. There are differences depending on the particular area; however, the dorsal stream is characterized by a more complete coverage of space including the visual periphery, with larger receptive fields, and foveal sparing in some instances. In contrast, areas of the ventral stream show greater focus or coverage of visual space in close proximity to the fovea.

We propose that the segregation of dorsal and ventral visual pathways is fundamentally grounded in the difference in representation of visual space of the two pathways (Figure 2): Cortical areas of the ventral visual pathway emphasize central vision, with neuronal receptive fields in and around the fovea; in contrast, cortical areas of the dorsal visual pathway emphasize complete vision, with neuronal receptive fields distributed across nearly all of visual space and less intense focus, in comparison with areas lying in the ventral visual pathway, on the fovea. As we argue below, the difference in the representation of visual space between the two pathways is the principal driver underlying functional differences between them.

We briefly describe some of the evidence in support of the proposal. While there have been qualitative reports of measurements of receptive field properties in a number of cortical areas over the years, for our purposes, we will focus on systematic studies that used quantitative measures using a hands-off computer based procedure. These studies, summarized in the two paragraphs below, show that for several cortical areas belonging to the ventral visual pathway in monkey and human, emphasis is placed on the processing of the fovea and nearby, whereas for cortical areas of the dorsal pathway, emphasis is placed on the processing of all of visual space.

The ventral posterior area (VP, also called ventral V3 or V3v/ventrolateral posterior area) is an important area in the ventral visual pathway. Calculations of cortical magnification as a function of eccentricity based on human fMRI data and equations given in Sereno et al. (1995) show that the cortical magnification factor decrease from fovea (2°) to periphery (10°) is 11.6% per degree of visual angle (assuming approximately linear decrease in magnification). Similar calculations for human area V4v yield a value of 11.3% per degree of visual angle increase in eccentricity. By comparison, the corresponding value for V1, which is well-known to have an overrepresentation of the fovea, is not higher: 10.8% per degree of visual angle increase in eccentricity; note that higher values indicate sharper fall-off in cortical territory devoted to more peripheral eccentricities and therefore, greater concentration of resources to central vision. Thus, the values for areas VP and V4v in humans show a high level of overrepresentation of the fovea compared with that of the rest of the visual field. fMRI studies on macaque monkeys yield similar results but with a different (but related) measure called visual field eccentricity which has a single free parameter called the scale factor that provides a scaled value for the amount of cortical space used to represent a range of visual eccentricities of visual field (usually 0–12°): higher the scale factor, lower is the cortical magnification (Brewer et al., 2002). The scale factor for macaque area V3, which includes area V3v, is 0.08 (by comparison, the scale factors for monkey areas V1 and V2 are 0.06, which shows how close V3v cortical magnification is to that of V1 and V2; in human area V1, the scale factors are 0.03–0.035, about half of that seen in monkeys, indicating that humans have about double the cortical magnification). A study examining topography in V3 and V4 (Gattass et al., 1988) fitted cortical magnification as a function of eccentricity with power functions and found slopes of −0.74 and −0.90 for V3 and V4, respectively (note that no overrepresentation of fovea is a slope of 0, and more negative values indicate greater overrepresentation of the fovea), again showing significant overrepresentation of the fovea in cortical areas V3 and V4 of the ventral visual pathway. A cluster of higher-order areas belonging to the ventral visual pathway is found in the inferior temporal cortex (IT). Neurons in IT have large receptive fields, and yet they show a preference for foveal positions. Op De Beeck and Vogels (2000) provided the first detailed, quantitative data on the spatial sensitivity of neurons in monkey area TE (anterior part of IT) and found that overall, TE neurons showed a clear bias for responding most strongly to stimuli presented in the fovea or near the fovea (4° eccentricity). Further testing with low-pass filtered versions of the stimuli revealed that the general preference for the foveal position remained and therefore, was not due simply to TE neurons receiving input with a lower spatial resolution at more eccentric positions. Earlier, qualitative reports in anesthetized (Gross et al., 1972; Desimone et al., 1984; Kobatake and Tanaka, 1994) and awake (Schwartz et al., 1983; Komatsu and Ideura, 1993; Tovee et al., 1994; Missal et al., 1999) monkeys converged to the same general conclusion: neurons in TE respond most strongly to stimuli in the foveal position and prefer stimuli presented in the contralateral hemifield. fMRI recordings from monkey area TEO, also in IT, yielded a weak signal, so quantitative measures were not possible (Brewer et al., 2002); however, the authors stated that the central visual field occupies a large proportion of the cortical area, making it harder to define an eccentricity map. Related to the greater focus on central vision in the ventral visual pathway is the sensitivity to retinotopic information in ventral visual areas. It is well-known that areas V3v, V4 have retinotopy, but, more surprisingly, sensitivity to retinotopic information is observed in neurons of macaque area IT (Lueschow et al., 1994; Op De Beeck and Vogels, 2000; DiCarlo and Maunsell, 2003). Lehky et al. (2008) states, “With respect to retinal position, about two-thirds of AIT neurons were sensitive to modest shifts in the retinal stimulus location. While from one perspective this retinotopic modulation is simply a manifestation of the existence of a receptive field, from another perspective it indicates that inferotemporal cortex retains information about the spatial position of objects in retinocentric coordinates.” Lehky and Sereno (2011) built on these findings in a model that had IT neurons with realistically large receptive fields, nonetheless had exquisite position selectivity and localization.

We contrast the above results in the ventral visual pathway with results from recordings of neurons in the dorsal pathway: Quantitative measurements of receptive fields of cortical areas in the dorsal pathway using automated techniques similar to those used to characterize ventral visual areas yielded far lower proportions of neurons with receptive field centers on or near the fovea. Our analysis of the literature leads us to estimate that ~14% of MT neurons (29/213 neurons illustrated in Figure 6 of Raiguel et al., 1995) and ~7% of MST neurons (6/85 neurons illustrated in Figure 2 of Raiguel et al., 1997) recorded have receptive field *centers* within 5° from the fovea; ~35% of MT neurons (75/213 neurons from Raiguel et al., 1995) and ~20% of MST neurons (17/85 neurons from Raiguel et al., 1997) recorded have receptive field centers within 10° from the fovea^2^. Quantitative analysis of monkey area MT leads to an estimate of visual field eccentricity scale factor of 0.10 (by comparison, the scale factor is 0.08 for areaV3v, which is 20% smaller; Brewer et al., 2002). Nonetheless, other studies in the macaque claim that the fall-off in cortical magnification with eccentricity in MT (Gattass and Gross, 1981) is comparable to V1's (Gattass and Gross, 1981; Gattass et al., 1981). Thus, MT may well be an important exception to our overall argument, although it should be pointed out that said study (Gattass and Gross, 1981) found no MT RF *centers* at a distance closer than 5° from the vertical meridian (they found a somewhat crude topography in MT, i.e., scatter in MT RFs at a given eccentricity is high and is the result of the large RF sizes of MT cells). Area V6 has been studied in monkey and in human and Fattori et al. (2009) state that what is special about V6 “…is its lack of a ‘magnification factor,’ that is of an overrepresentation of the central part of the visual field,” and rather uniformly represents the visual field. One of the salient characteristics of V6 topography, the authors note, is that V6 represents the whole contralateral visual field. The authors further claim that previous fMRI studies failed to find V6 in humans because they stimulated the central 8–12° of the visual field, and not the periphery; in contrast they stimulated the entire visual field up to 110° in total visual extent and were successful in locating the human analog of V6. Using an automatic receptive field mapping procedure with white noise stimulation and reverse correlation techniques similar to those used in the studies described above (Raiguel et al., 1995, 1997), Ben Hamed et al. (2001) found that across the population of neurons recorded from area LIP, there was an overrepresentation of foveal space relative to peripheral space (22% of cells recorded had receptive field centers of mass within the central 3° of visual field, 40% in the central 5°, ~50% in the central 6°, and 70% in the central 10°). The cortical magnification factor decrease in resources from fovea (2°) to periphery (10°) is a modest 4.5% per degree of increase in eccentricity of visual field (assuming approximately linear decrease in magnification). Using a similar set of techniques and measurements, the authors found that area VIP, also an area of the dorsal visual pathway immediately adjacent to LIP, exhibited negligible overrepresentation of foveal space, and far less than that in LIP, with 50% of VIP neurons representing a region extending up to 20° of visual field.

In summary, the ventral and dorsal visual pathways, with a few exceptions, place different emphases on the coverage of visual space and in the degree of visual topography. Others have concluded as much (see e.g., Gattass et al., 2005), but here, we further state that the underlying difference in visual coverage in the two pathways underpins functional differences between them, e.g., the preferential involvement of the ventral stream in object recognition and of the dorsal stream in motion processing and sensorimotor control (see below).

**Figure 3:**
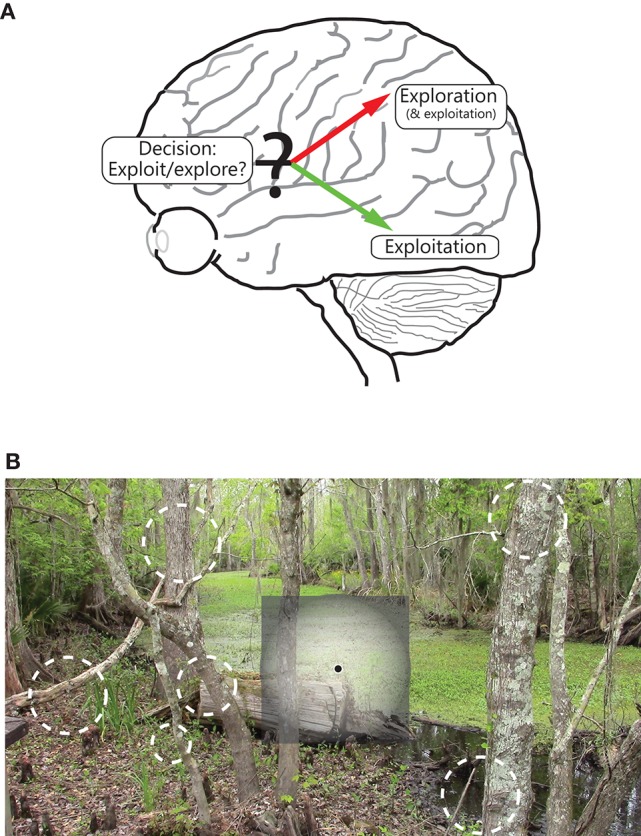
**Illustration of the functional segregation of the dorsal and ventral pathways. (A)** Shows the following: The dorsal pathway is engaged in exploration, and to a certain extent, exploitation, of the environment, whereas the ventral visual pathway is engaged in exploitation of a focused part of the environment. As illustrated, the frontal areas are engaged in decision making, including on deciding between whether to exploit or explore. **(B)** shows how the two pathways process space, namely while the ventral (and to a lesser extent, dorsal) pathway emphasizes the central part of the visual field, the dorsal stream emphasizes all of visual space; salient, moving and/or changing parts of the scene in the visual periphery are vying for the observer's focus and attention, and decisions are being made on where in space to move the eye.

We further propose that the difference in visual coverage between the two visual streams can be interpreted functionally as exploitation (ventral stream) vs. exploration (dorsal stream) of the external environment (Figure 3A). At the most basic level, the environment consists of a unitary biologically or behaviorally relevant stimulus that the organism is focused on while allocating maximum cortical resources and highest resolution apparatus into gathering detailed information about it (exploitation), whereas other stimuli scattered over space are competing with each other and with the currently focused stimulus for the organism's attention (exploration); these stimuli are processed crudely, not enough to reliably identify them or to process in minute detail, but just enough to acquire the information required to inform decisions on where next to move one's gaze and/or attention.

The two functions map one to one onto the differing spatial properties of the two pathways. Cortical areas that have greater spatial resolution, and more ordered topography, are better suited to extract high quality, structured information about a stimulus that is at the center of focus (note the extent of coverage of visual space matters little for its successful implementation, and covering all of visual space at high resolution is impractical and expensive anyway). The ventral stream is well-suited for this purpose (Figure 3B). On the other hand, cortical areas that coarsely process most of visual space are better suited to fully explore the external world, detect novel, dynamically changing stimuli occurring anywhere in space, and execute plans to bring one (or more) of them into focus for further, more detailed processing. The dorsal pathway is well-suited for this purpose (Figure 3B)^3^.

The proposed functional dichotomy circumvents at least three of the issues that plague past models of visual function. First, exploration of the environment depends, at a very fundamental level, on the capacity to deploy resources to the entire environment—which, as discussed above, areas of the dorsal stream with their ambient coverage of the visual field possess—, the ability to select salient, biologically and/or behaviorally relevant items from the environment, and the resources to pull one's attention and gaze toward them in order to bring to bear the high resolution, but metabolically expensive, apparatus to acquire better, high quality information. These mechanisms fall under the purview of spatial attention *ipso facto*, which several investigators have claimed is a crucial function of the dorsal pathway. Second, the proposition that the ventral visual pathway has a broader function beyond object vision follows rather straightforwardly from the proposed framework: Exploitation of visual information is to obtain the best quality information possible about the stimulus in focus, and the data acquired will inevitably contain information on various features of said stimulus (or multiple stimuli within the focus), and not just pertaining to objects. Third, the crucial but limited role of the dorsal stream in sensorimotor control of objects in the periphery (and a role in spatial vision) falls out of our proposed framework. In comparison with the ventral visual pathway, the dorsal pathway places greater emphasis in processing the visual periphery (note: the dorsal pathway processes the center as well); its corresponding function of scene exploration and associated role in bringing items that lie in the periphery into central focus for further processing implies greater involvement of the dorsal stream in sensorimotor control^4^. Similarly, the emphasis on coverage of a more complete visual field in the dorsal pathway lends said pathway to be more conducive to locating stimuli (see e.g., Lehky et al., 2013; Sereno et al., 2014 for an elegant computational argument favoring spatial localization of stimuli by areas of the dorsal stream), visuospatial perception—especially involving spatiotemporal integration over multiple fixations or position estimation with respect to a frame of reference (see e.g., Pisella et al., 2013a showing individuals with lesions of the dorsal stream perform poorly on elementary visuospatial tasks such as line bisection and tests consisting of comparing the positions of dots within a frame), the identification of large objects involving the integration of information over time and space (e.g., across multiple fixations or relative to a landmark or frame of reference, for which the large receptive fields of cells in the dorsal stream are better-suited and in which stable representations integrating spatial memory and on-line information are formed), and *de novo* learning of novel perceptual classifications (see *Perceptual classification and the two streams* below).

Furthermore, the proposal provides the beginnings of a foundation with which “…to address the dynamic details of how the many visual brain areas arrange themselves from task to task into novel functional networks (McIntosh and Schenk, 2009).” In real life, with a seemingly unending barrage of salient *and* novel stimuli in the environment, the transition from exploitation to exploration, or vice versa, is often seamless, and switches in behavioral mode occur multiple times over a short period. One expects continual tension between the two functions—a kind of yin-yang—and constant interaction between the two pathways while the organism engages with the environment. Information about a salient stimulus at the center of attention and gaze is gathered while novel stimuli away from the focus vie for attention and gaze. The relative, continuously changing internal weights of the various stimuli—those that are within the focus of attention and gaze and those that are not—dynamically determine the see-saw between exploitation and exploration, as well as the continually evolving interaction between the ventral and dorsal pathways (this can be interpreted as a neural variant of the exploration-exploitation dilemma; see e.g., Sutton and Barto, 1998). It is reasonable to speculate that the transition from exploitation to exploration, and vice versa, is governed by areas in the prefrontal cortex, i.e., the prefrontal cortex helps determine, on a moment-by-moment basis, the mode of visual processing. Thus, under our proposal, a role for the prefrontal cortex emerges that is in line with what is known about the general, well-established role of the prefrontal cortex in decision-making (see e.g., Krawczyk, 2002, and references therein). In contrast to the present model, it is difficult to argue, on the basis of past models, for when and how transitions from processing “what” an object is to “where” an object is, or vice versa, might occur: one must know where an object is first before knowing what it is; on the other hand, one could argue that one must know what an object is in the first place before understanding where it is. This is a classic chicken and egg problem. Consequently, a scheme based on the processing of objects fails in providing a roadmap for understanding how the two visual pathways would interact in common situations. Similar logic applies to the action/perception model. When do we use vision to perceive and when to act? And why can we not perceive while we act or act while we perceive? These are questions for which answers are not easy to intuit. In this context, it is important to point out that in our proposal, the interaction between the ventral and dorsal pathways is not necessarily always a competitive one. Under impoverished stimulus conditions, or given high task demands, information from both streams is likely to be pooled for improved processing. Depending on the familiarity and difficulty of the task and how specialized the information required is, the two streams may interact either competitively or cooperatively; the details of the interaction remains an open question for further research.

It is important to caution against those that argue that our proposed functional dichotomy is iron-clad. Differences between dorsal and ventral streams are a matter of degree rather than a strict division of labor. In particular, the dorsal pathway processes information from the entire visual field—including the visual center—and therefore, can, and does under circumstances described below, also subserve the function of exploitation. An arguably more complete summary would be that while the ventral visual pathway subserves exploitation, the dorsal pathway does both: exploration and, at times, exploitation (see e.g., Ben Hamed et al., 2002) in which the cells of area LIP in the dorsal pathway of the monkey exhibit smaller receptive fields shifted toward the visual center in the attentive fixation condition and, in contrast, receptive fields not shifted toward the center in the free gaze condition; studies showing extraction of depth structure and discrimination of 3D shape in several areas of the parietal cortex in the monkey Durand et al., 2007, 2009). We investigate this issue in the context of perceptual classification below.

## Perceptual classification and the two streams

Perception is arguably the end product of a categorization process (Bruner, 1957), and categorization is a significant outcome of sensory information processing by the cortical pathways. Studies of perceptual classification and the role of cortical areas of the dorsal stream described in the following paragraphs argue in favor of the idea that the dorsal stream can subserve similar functions as the ventral stream.

On a task involving the detection of a target that was a conjunction of color and direction, neurons in area LIP of an over-trained monkey exhibited selectivity to direction as well as color (Ibos and Freedman, 2014), which is a feature not typically associated with the dorsal stream. A different study arrived at a similar conclusion: monkeys were trained to covertly search for targets defined by a unique conjunction of color and motion features and to signal target detection with an eye movement to the putative target; recordings from directionally selective cells in visual area MT revealed the emergence of selectivity for color and modulation of response by color (Buracas and Albright, 2009). In a different study altogether, when a primate was trained on categorization of motion directions into two arbitrary classes, i.e., the learning of a novel stimulus-response association, MT neurons, which are natively direction selective to begin with, showed sharp differences in response to different directions, but hardly any category based differences, unlike LIP which are not natively direction selective but exhibited sharp category based directional differences in firing rate following the training (Freedman and Assad, 2006).

The studies above all have the following elements in common: a primate undergoes extensive training in the laboratory to learn an arbitrary perceptual classification based on a visual feature or combination of features; the over-trained monkey now performs at a high level of accuracy on said classification behavior; single-unit recordings reveals cells in a cortical area that respond to and exhibit some degree of tuning for a feature that said area in the naïve animal was not known to be tuned for.

We argue that the emergence of tuning for a particular visual feature in the cortical areas cited above and others described below is the result of a refinement of latent but weak capacity already present in these areas—a refinement that arises from intensive training lasting several months to a year. The latent capacity may be due, in part, to projections from areas, which often lie in the ventral visual pathway, already selective for said features to areas in the dorsal pathway. These connections are presumably enhanced during the course of the intensive overtraining, e.g., studies have revealed connections in both directions between V4 and MT and between V4 and LIP (Ungerleider et al., 2008), which could explain the emergence of color tuning in LIP and MT in the aforementioned studies. We further speculate that the greatest capacity for plasticity related to perceptual classification may not be in the areas whose neurons already have hard-wired, refined tuning for the features that drive the novel, arbitrary classification behavior, but rather in the areas that have some innate response to the feature but not a well-tuned one (on a somewhat related note, experimental studies have found that cells and populations whose selectivity is displaced from the feature value used for the classification achieve the highest levels of discriminability for the classification, not those whose selectivity peaks at the feature value used for the classification (see e.g., Wilson and Regan, 1984; Vogels and Orban, 1990; Purushothaman and Bradley, 2005). Indeed, LIP neurons that were not direction selective during passive viewing in Ibos and Freedman (2014) showed larger directional tuning shifts during the delayed conjunction matching task (delayed match to sample task), i.e., natively directionally untuned neurons in LIP showed more change in directional tuning than natively tuned neurons, consistent with the point above. Our proposal of areas of the dorsal pathway acquiring tuning for features such as color and shape can be thought of as an extension of the above single-unit findings to brain areas^5^).

Other experimental findings from the primate literature are consistent with our proposal: Inferior temporal cortex has been known to process information about shape and yet, studies of visual-shape categorization (where the animal has to learn a new arbitrary perceptual classification of shape) comparing activity in prefrontal cortex and inferior temporal cortex found stronger signals for the newly acquired shape category in prefrontal cortex vs. IT (Sigala and Logothetis, 2002). The results are consistent with the idea that visual categorization for naturally occurring categories may be hard-wired in IT with little scope for plasticity in terms of learning new arbitrary categories based on shapes/features (what's more, it may even be detrimental to overturn perceptual classification acquired during development and more preferable to engage neurons in other areas to learn the new, arbitrary perceptual classification). On the flip side of the coin, the ventral visual pathway is also capable of similar feats of flexibility in acquiring tuning for visual features that the dorsal pathway is otherwise known for: Monkeys had to perform a difficult visual short-term memory task: they performed a delayed match-to-sample task using direction of motion as the matching criterion (Ferrera et al., 1994). Note that cells in MT and MST are more sensitive to motion direction than V4 in the ventral visual pathway, and yet the direction of the remembered sample modulated the response of V4 neurons more than the responses of neurons in MT, MST, and area 7a—all in the dorsal pathway; across the population of cells recorded in each cortical area, the size of the modulatory effect was in the following order: V4 > 7a > MST > MT. The result was in sharp contrast to measures showing that in terms of selectivity for motion direction of the sensory stimulus, the order was MT > MST > 7a > V4^6^. As before, these results and the ones cited in the paragraphs above affirm the proposal that it is not the cortical area that is tuned to the processing of a particular visual feature that shows sharp task-based tuning for said feature in a behavioral, goal-directed context, but rather an area that has weak native tuning for said feature (irrespective of whether the area is in the dorsal or ventral stream). In a narrower sense limited to the present proposal, the primate studies on perceptual categorization reveal that the dorsal pathway can become involved in the fine processing of visual features, shapes and objects, i.e., functions associated typically with the ventral visual pathway, when an individual is trained intensively on a task to learn a novel, arbitrary stimulus-response behavior/association utilizing these features.

The transient, task-based acquisition of (sometimes sharp) tuning for visual features by areas of the dorsal pathway is not inconsistent with the idea that these areas may not always be utilized to perform the task in question at all. The area has to be incorporated into the pathways used to perform the trained task and the brain has to learn *de novo* to *involve* the given area in said task. There have been recent, surprising reports of areas emerging with exquisite tuning for a particular feature from extensive task-related training but no involvement of the area in the task, i.e., no effect on task performance driven by the newly acquired feature from removal of the area (Chen et al., 2016; Katz et al., 2016). In line with this argument, inactivation of the LIP was found to have little effect on non-spatial aspects of decisions, including sensitivity to reward or the ability to switch preference upon reversal of reward contingencies (see Figure 4C of Balan and Gottlieb, 2009). The findings suggest that the non-spatial responses in LIP do not indicate direct involvement in non-targeting manual actions; instead, they appear to be feedback signals related to the selection of a relevant location. As stated in Gottlieb and Snyder (2010), the non-spatial feedback found in the parietal lobe may reflect, in part, this type of computation, through which the brain identifies stimuli that are associated with—and thus can predict—other variables of interest such as an action, rule or expected reward. As Gottlieb and Snyder (2010) summarize, “Despite the ubiquity of non-spatial information in individual neurons (of the dorsal pathway), reversible inactivation of the parietal lobe affects only spatial orienting of attention and gaze, but not non-spatial aspects of performance.”

As mentioned above, the dorsal pathway is likely to have a role to play in the processing of items in fine detail—at least under conditions that favor its use. Patients with optic ataxia, which arises from damage to the parietal cortex, show a clear deficit in locating the mid-point of a line (line bisection task) only when big lines are shown; they show no deficit when asked to bisect very small lines (Pisella et al., 2013a). This selective deficiency indicates a role for the parietal cortex in integrating space during active ocular exploration. It is likely that the dorsal stream, with neurons with large receptive fields, areas that cover large expanses of space, and input from multiple sources (e.g., eyes) and spatial memory, is useful when it is required to integrate information from a large area (or in the control of visually guided actions like reach-to-grasp movements involving the extraction of 3D shape). Extracting depth structure from large planar surfaces may be one such example of this requirement. In line with this, it has been shown that many neurons in area CIP, an area in the anterior region of the parietal cortex prefer very large 3D surfaces (Shikata et al., 1996). Filling the surfaces of objects defined by their 2D shape outlines was found to induce additional activation in regions LIP and AIP of parietal cortex over and above that elicited by the outlines themselves (Durand et al., 2007). These results suggest that said areas respond more to 2D shapes (grayscale images) than simple outlines/contours (and to recovering depth structure of 3D objects), although a high degree of selectivity for specific shapes over others, or for certain categories of 2D and 3D objects over others were was not shown in the said study^7^. Note that determining depth structure necessitates integration over time and space. The results are thus consistent with our assertion that areas of the dorsal pathway are well-suited for form discrimination over large areas of visual space, often involving integration over multiple retinal images across time and space.

A role for the dorsal pathway in the function of object recognition—and other assorted non-spatial functions—can be thought of as an extension of the theme of exploration that we proposed above. The proposed role of the dorsal pathway in exploration of the environment is in finding which items, what class of items, or where in space to next process and learn about in greater detail. The role is therefore likely to be modulated by saliency and reward: Items that are associated with a reward (e.g., a drop of juice), or that are considered salient^8^, will be explored first and queued up for exploitation next. Modulation of the response of cells in the dorsal pathway by reward follows from the above argument (see e.g., (Peck et al., 2009) showing that reward associations modify the bottom-up salience of a visual stimulus independently of a motor output in posterior parietal cortex). Similarly, determination of object identity can be an intermediate step toward achieving the goal of exploration (see e.g., (Ibos et al., 2013) where recordings of LIP and FEF cells in overtrained monkeys reveal the emergence of cue identity and cue location prior to attention cueing).

## Multisensory processing and the visual cortex

Studies of multisensory integration in single cells of the superior colliculus of mammalian species have yielded the principle of inverse effectiveness (Meredith and Stein, 1983, 1986), which is that multisensory integration is more effective when the constituent stimuli of a single modality evoke relatively weak responses. In modern parlance, the principle is the result of a Bayesian approach to cue combination. It is not too far a reach to extend the principle, which originated from observations of single cell recordings, to brain areas: Sensory cortical areas in which neurons exhibit responses to a second modality—and are therefore termed multisensory—appear to show responses to the primary modality that are usually less precise and noisier than responses of neurons in unimodal counterparts. For instance, neurons in auditory cortical regions immediately caudomedial to macaque A1 (in the belt region of the auditory cortex in the superior temporal plane) display broad auditory frequency tuning curves, i.e., low precision in response and band-passed noise responses larger than pure tone responses; these regions also have robust somatosensory responses co-represented with auditory responses. In contrast, no somatosensory responses were found in A1 (Schroeder et al., 2001).

Here, we extend the principle further to visual processing streams. Multisensory integration is overall more effective in the dorsal than in the ventral pathway. We begin by noting that damage to different areas of the dorsal pathway lead to problems with the processing of non-visual modalities and integration with vision, with the problem depending on the exact site of damage, i.e., sub-pathway in parietal cortex (Pisella et al., 2006), e.g., unilateral optic ataxia patients exhibit reach-and-grasp errors when the object is presented in their contralesional visual field with either hand (“field effect”) and when they use their contralesional hand toward either hemifield (“hand effect”); errors linked to the hand effect appear to be related to a mislocalization of the contralesional (ataxic) arm based on impairment of high-level processing of proprioceptive information (Blangero et al., 2007) (Interestingly, movements toward objects performed in central vision are preserved especially when they are performed with vision of the hand, which compensates for the proprioceptive mislocalization deficit). We further note that whereas multisensory processing (single-cell and areal convergence) is observed in both pathways, multisensory neurons and multisensory areas are more commonplace in the dorsal pathway. Multisensory convergence has been demonstrated in posterior parietal and ventral intraparietal regions of macaque neocortex (Hyvärinen and Shelepin, 1979; Duhamel et al., 1998; Andersen and Buneo, 2002), which are a part of the dorsal stream; the superior temporal sulcus and temporal-parietal association areas (Benevento et al., 1977; Leinonen et al., 1980; Bruce et al., 1981), which are commonly accepted as not part of either stream; premotor cortex and area 6 of the frontal cortex (Rizzolatti et al., 1981; Graziano et al., 1994), which are areas to which the streams converge. In the human, imaging yields a similar pattern: Besides the insula (Gentile et al., 2011) and superior temporal sulcus (e.g., Calvert et al., 2000; Nath and Beauchamp, 2011, 2012), the following areas of the dorsal stream—right temporo-parietal junction, intraparietal sulcus (e.g., Calvert, 2001; Gentile et al., 2011) and more broadly, the posterior parietal cortex (Pasalar et al., 2010; Huang et al., 2012), and human MST (e.g., Beauchamp et al., 2007) have been shown to participate in multisensory processing. (As mentioned above, multisensory integration and convergence is not altogether absent in the ventral visual pathway: The perirhinal cortex, or PrH, in the ventral visual pathway is a multisensory area that participates in object recognition (Lehky and Tanaka, 2007). Single cell and multi-unit recordings (Desimone and Gross, 1979) in macaque revealed cells in PrH that were responsive to visual, auditory and somatosensory stimuli, and cells with very large RFs, even larger than in TE, often approaching the size of the animal's visual field. However, multisensory information is used by the PrH not for perceptual ends but conceptual ones: PrH integrates crossmodal features into high level *conceptual* representations defined by crossmodal binding of stimulus features (Taylor et al., 2006), so that integrating sounds with congruent visual stimuli (e.g., knowing to integrate a meow with the picture of a cat, but not with a picture of an elephant) based on semantic memory relies on PrH. Patients with lesions, including the PrH, but not patients with damage restricted to frontal cortex, were impaired on the same crossmodal integration task, and their performance was significantly influenced by semantic factors.) In short, there is a preponderance of cortical areas in the dorsal visual pathway receiving multisensory input. More specifically, visuo-tactile and visuo-proprioceptive multisensory convergence and integration are ubiquitous in the dorsal pathway even though audiovisual multisensory convergence and integration is not^9^ [but see (Skipper et al., 2007), which showed significantly greater activity in the inferior parietal lobule and in the precuneus—a part of the superior parietal lobule for the perceived audiovisual (McGurk-MacDonald) syllable vs. other audiovisual syllables].

A simple extension of the inverse effectiveness principle suffices to account for the difference in multisensory convergence of the two streams. Larger receptive fields of neurons in areas of the dorsal stream, their relatively greater emphasis on processing the visual periphery, and relatively weaker tuning for features overall are consistent with poorer quality of visual processing and greater uncertainty about feature value (and perhaps about spatial location as well—but see Lehky and Sereno 2011 for a computational counter-argument). Apropos, it has been shown that the responses of neurons in cortical area LIP of the dorsal stream have larger Fano factors, namely greater trial to trial variability in response to the same stimuli than neurons in area AIT of the ventral stream (Lehky and Sereno, 2007). Bayesian statistics model the integration of information from multiple sources by the brain relatively well: with greater uncertainty of information from one source—in the present case, visual— information from additional sources is integrated more. The final perceptual decision is weighted by the reciprocal of variance of each source. So that noisy sources carry less weight; the Bayesian process minimizes the variance or noise in the final estimate of the feature being discriminated or judged. The procedure of cue combination known as maximum likelihood estimation has been observed in several studies of sensory information processing in the human (Clark and Yuille, 1990; Blake et al., 1993; Landy et al., 1995).

Using similar logic, information from multiple sources, e.gs., signals from both eyes, signals from multiple modalities over and above vision, is likely to be incorporated more into the spiking responses of neurons and areas of the dorsal vs. ventral stream. Recent studies of patient DF, the patient with ventral visual form agnosia and relatively (though not entirely) intact dorsal stream, confirm that DF has normal grasping abilities, but that these skills decline sharply when haptic feedback is removed (Schenk, 2012), indicating that visuomotor control—presumably under the control of the dorsal stream given the stimulus conditions of the study—arises from multisensory interactions. Patients with visual form agnosia have profound deficits in their otherwise relatively normal grasping when binocular information is removed (Keefe et al., 2011), because the dorsal stream exploits the redundancy available in multiple sources of information, and integrates binocular and monocular cues to improve grasping performance, consistent with cue integration theory. Dependence on a range of (retinal and extra-retinal) cues from multiple sources is thus a hallmark of behaviors that critically engage the dorsal stream. Apropos, a few areas of the dorsal pathway receive multiple sources of subcortical input. Area MT of the dorsal stream receives input from the LGN, both via cortical areas V1 and V2 and directly as well (Sincich et al., 2004), and from the superior colliculus via the pulvinar. The direct LGN projection to MT is in line with the Rosa and Tweedale's (Rosa and Tweedale, 2005) assertion that MT should be considered a primary visual area, in line with V1; we extend their argument by stating that MT can be thought of as a primary visual area for the dorsal pathway. The pulvinar does receive and send projections to both areas of the dorsal, i.e., posterior parietal cortex and (ventro-lateral pulvinar) ventral streams, e.g., V4 and IT. However, as has been summarized in Zhou et al. (2016), the ventral cortical pathway leads the pulvinar in response, thereby arguing for a stronger corticopulvinar projection. Moreover, as the authors argue, the ventro-lateral pulvinar, which connects with V4, is not a major source of feedback that directly mediates the effects of attention in the cortex. In contrast, there is strong evidence that the dorsal portion of the pulvinar with connections to the dorsal stream (Baizer et al., 1993) has a role in attention similar to that of the parietal cortex. Deactivation of the dorsal pulvinar impairs attention in a manner similar to parietal deactivation (Robinson and Petersen, 1992; Wilke et al., 2010). Indirectly, the results argue for a stronger connection from the pulvinar to the dorsal vs. the ventral stream although more direct studies measuring evoked responses are required. In sum, multiple sources of input are supported in the lower areas of the dorsal pathway and the dorsal pathway is better suited to take advantage of the multiple sources. Convergence from multiple modalities, or, more generally, multiple sources of information, i.e., modalities besides vision, information from both vs. a single eye, or information from more than one low-level visual source downstream, is somewhat more common in the dorsal pathway.

## Why the dorsal stream is better-suited for the processing of motion

Cortical areas of the dorsal stream contribute to motion perception. Neurons in cortical areas of the dorsal stream exhibit clear direction selectivity (area MT: Dubner and Zeki, 1971; Albright, 1984; Albright et al., 1984), are important for the execution of smooth pursuit eye movements and in the analysis of optic flow elicited by self-motion (area MST: Desimone and Ungerleider, 1986; Tanaka et al., 1986; Duffy and Wurtz, 1991; Bradley et al., 1996), and show greater response to drifting bars than to flashed spots (area MT: Allman and Kaas, 1971); individuals with damage to these areas suffer from akinetopsia, i.e., visual motion blindness (Rizzo et al., 1995; Zihl and Heywood, 2015)—the selective inability to perceive visual motion and the persistence of strobelike images instead, or the Zeitraffer phenomenon (Ovsiew, 2014)—the altered perception of the speed of moving objects. Here, we offer an account for the preponderance of the dorsal stream in the processing of visual motion that follows from the dichomotous scheme we outlined above.

**Figure 4:**
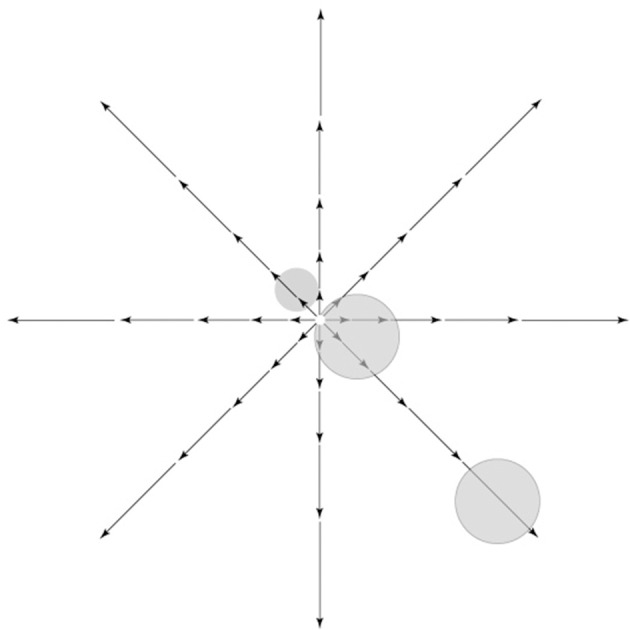
**Retinal image motion, called optic flow, induced by self-movement in a structured environment**. Vector lengths represent strength of the motion signal as a function of eccentricity. As shown, there is zero motion at the center of gaze in a radial optic flow field, while strong motion input is present away from the center of gaze, namely in the visual periphery. Receptive fields of hypothetical cells near the fovea or in the periphery are shown on top of the optic flow pattern.

Self-motion generally involves movements toward or away from an object. In natural environments, movements toward prey or movements away from predators are prevalent, and, in general, goal-directed actions toward or away from an item in the environment (toward food, drink, a potential mate or escape from a perceived threat) are likely to constitute an important, arguably dominant, behaviorally relevant class of movements. Radial optic flow patterns are generated as a result of these self-motion trajectories and their importance is indicated by the overall greater responsiveness of the set of cortical areas in the IPS to radial, as compared to planar or circular, flow fields (Silver and Kastner, 2009). Several areas in the dorsal stream of the macaque, i.e., V6 (Fan et al., 2015), VIP (Chen et al., 2013), and MSTd (Lee et al., 2011) have been found to process information related to self-motion and heading. Radial optic flow patterns have a curious property—as Figure 4 shows, there is zero motion at the center of gaze in a radial optic flow field (termed the focus of expansion, or FOE), while strong motion input is present away from the center of gaze, namely in the visual periphery. One of the cornerstones of our proposed scheme is that the dorsal stream, unlike the ventral, extensively processes the visual periphery (in addition to processing the center). The above point dovetails nicely with the stark difference in motion input to the visual center vs. the visual periphery for a biologically significant class of motion signal^10^. Combined, they provide a rational basis for the greater contribution of the dorsal stream to the processing of visual motion.

## Why the dorsal stream is better-suited for sensorimotor control

Saccadic eye movements and arm movements such as pointing, reaching, and grasping constitute an arguably predominant class of goal-directed movements, and actions that involve fine-grained sensorimotor transformations requiring a high degree of precision and coordination amongst diverse muscle groups of different effectors.

**Figure 5:**
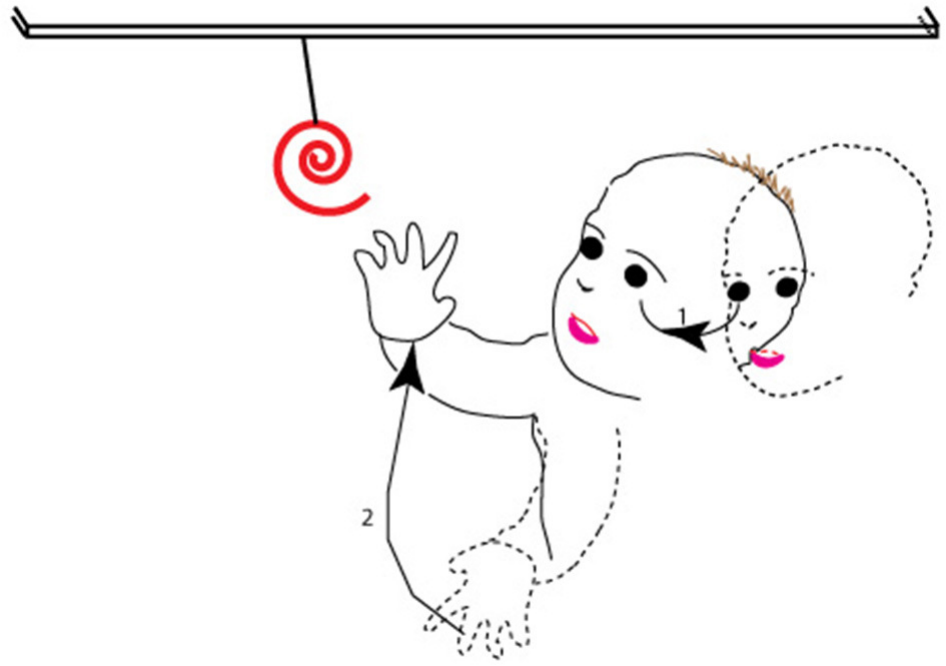
**The sequence of movements associated with a typical goal-directed behavior**. As shown, after spotting the target in the periphery (dashed outline of face), the observer moves his or her gaze to it (1 in the figure), so that at the end of the movement (solid outline of face), the target is at the center of gaze. The second action in the sequence (2 in the figure) is of the observer moving his hand from far peripheral, peripersonal space (dashed outline of hand) and shaping his/her fingers to align the hand and fingers with the target in the fovea, so that at the end of the second movement (solid outline of hand), the hand has moved to lie within the central field of view.

Here, we argue how the dorsal stream is naturally better suited to contribute to the planning and execution of these movements, and sensorimotor control in general, on the basis of the themes we have developed above. We remind the reader that goal-directed behaviors that involve the eye or arm comprise a sequence of actions. First, the observer locates the target in the environment—typically in the visual periphery. Then, the observer moves his or her gaze to it, which entails movement of the eye and head to a location in the visual periphery. In parallel, but after a delay which arises because of the greater inertia of the hand vs. the eye, the observer moves his/her hand to the recently foveated target and shapes his/her fingers in anticipation of the impending grasp; note that this is a movement of the arm and hand from peripersonal, peripheral space to the central field of view so as to align the hand with the target now in the fovea (Figure 5; see also Pisella et al., 2009 for similar arguments). That is to say, the hand movement is then modified on-line based on perifoveal reafference of the target when the gaze has been oriented (Prablanc et al., 1979; Gaveau et al., 2014).

As noted, the sequence of actions involves the movement of targets or effectors from the periphery to the fovea. From our proposal, the dorsal stream is responsible for ambient processing (see also Trevarthen, 1968) and areas of the dorsal stream show greater coverage of the visual periphery. Furthermore, goal-directed actions of the eye and hand typically engage the visual, proprioceptive, vestibular, and tactile modalities (Rossetti et al., 1995; Collins et al., 2000; Becker et al., 2002; Cordo et al., 2011; Blanchard et al., 2013). The coordination of information from multiple modalities requires multisensory neurons and areas. We argued above for the greater effectiveness of multisensory processes on the response and function of areas of the dorsal visual pathway. Several lines of reasoning thus converge on the proposition that the dorsal stream is better suited to the coding and execution of actions in our daily lives.

## Why the ventral stream is better-suited for object recognition and feature vision (whereas the dorsal stream is not unresponsive to mere flashes of light)

**Figure 6:**
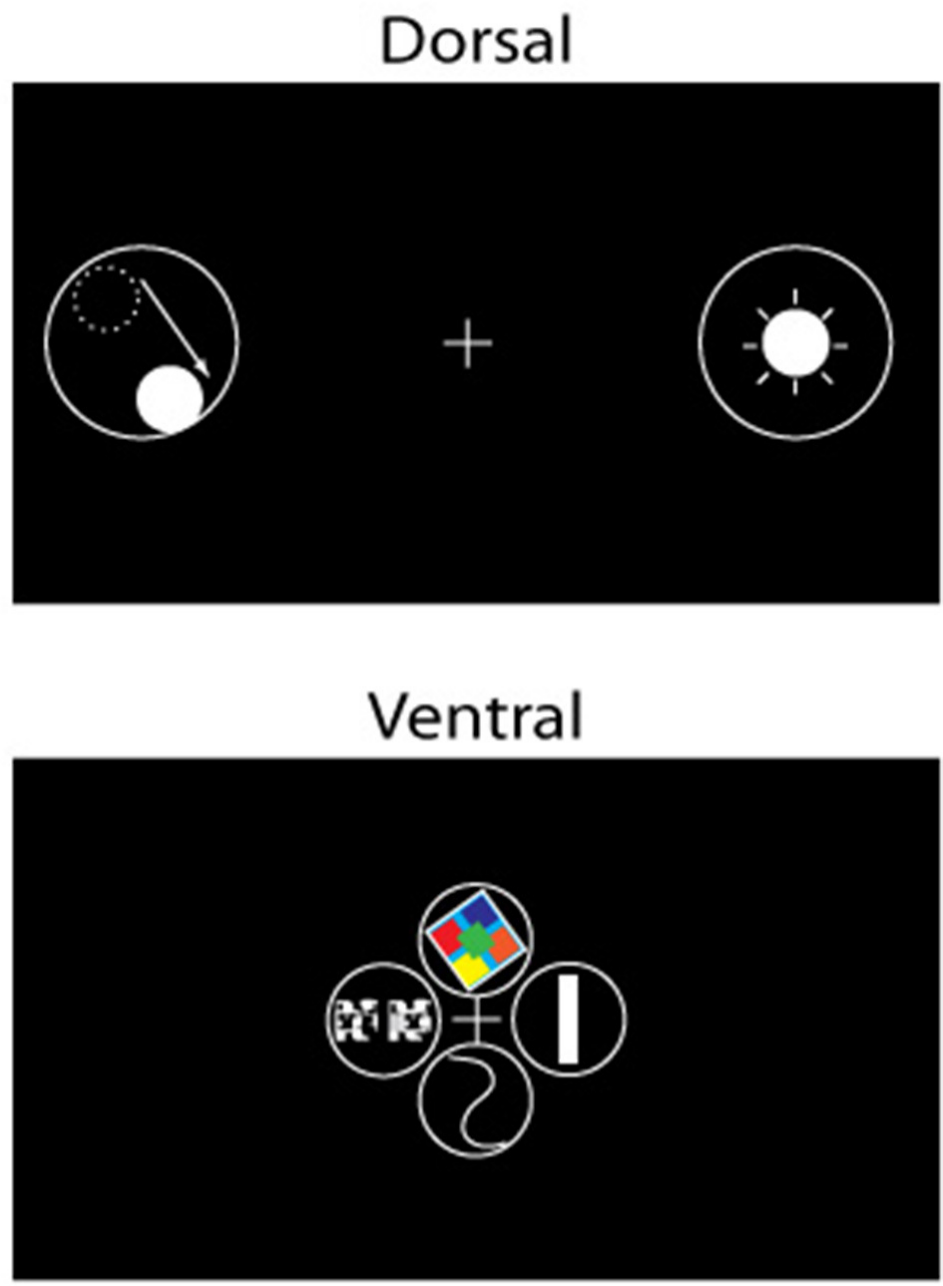
**Schematic illustration of the visual stimuli that elicit a strong response for cells (receptive fields represented by solid lines) in the dorsal (left) and ventral (right) visual pathways**. Cells of the dorsal pathway respond to flashing or moving spots of light in specific locations of visual space, whereas cells of the ventral pathway respond preferentially to visual features, e.g., (clockwise from top) color, orientation, shape, and depth, among others. The center of gaze is represented by a cross (+) in the figure.

As discussed above, neurons in areas of the ventral stream generally have smaller receptive fields than their corresponding counterparts in the dorsal stream. Smaller receptive fields mean finer-grained sampling of physical space, and available machinery for the processing of higher spatial frequencies, which are key to fine-level, subordinate categorization of objects (Note that the magnocellular and parvocellular projections from the retina to the ventral and dorsal streams differ in terms of sensitivity to spatial frequency content as well, again based on the relative sizes of the respective receptive fields of the retinal cells. The ventral visual pathway receives both magnocellular (sensitive to low spatial frequencies) and parvocellular (sensitive to medium to high spatial frequencies) inputs (Ferrera et al., 1992), whereas the dorsal visual pathway receives mainly magnocellular input (Merigan and Maunsell, 1993). Thus, complete information required for object categorization—both low and high spatial frequency (Collin and McMullen, 2005; Harel and Bentin, 2009)—flows into the ventral stream). Furthermore, receptive fields of cortical areas of the ventral stream are concentrated in and around the fovea, and the higher spatial resolution of central vision renders the ventral stream more suitable for the fine processing of objects as well as visual features (Figure 6)^11^.

Principles of self-organization argue further for the greater involvement of the ventral stream in feature vision. Cortical magnification of the visual center in the ventral stream, i.e., the disproportionately high number of neurons responsive to visual stimuli at and near the fovea, implies that there will be substantial overlap in receptive fields of neurons. As a result, when an external stimulus appears at or near the fovea, it will hypothetically evoke a substantial spiking response from a large sub-population of neighboring neurons in a cortical area of the ventral visual pathway (Figure 6). Even further, the presence of retinotopic maps in higher order areas in the ventral visual pathway in both non-human (Boussaoud et al., 1991; Rajimehr et al., 2014) and human (Larsson and Heeger, 2006; Sayres and Grill-Spector, 2008; Schwarzlose et al., 2008; Arcaro et al., 2009; Kravitz et al., 2010; Cichy et al., 2011) primates would mean that the appearance of a stimulus will cause a substantial part of the ventral visual pathway to potentially respond. However, biological and energetic constraints, including but not limited to the competition for limited metabolic resources (Wright and Bourke, 2013) preclude all neurons from firing at the same time, which occurs if neurons and areas specialize to inform on features over and above spatial location. In other words, metabolic constraints drive spontaneous symmetry-breaking along dimensions other than physical space, and the emergence and development of selectivity—at the single-cell level as well as the group or columnar level—for features such as color, orientation, objects, faces etc. across the cortex (Figure 6; von der Malsburg, 1973). Selectivity in feature space and object recognition in areas of the ventral stream are likely to arise from these constraints. The driving forces, i.e., cortical magnification, energetic constraints and retinotopy, are strong in the ventral stream; therefore, the development of feature selectivity and object recognition in areas of the ventral stream is more likely.

In sum, several rationales converge in arguing for the greater contribution of the ventral stream in feature vision, its greater involvement in object recognition, and in evaluating values of different features of the entity (object or part of an object or overlapping objects) currently on and near the fovea. The emphases and capabilities of the ventral visual pathway are in accord with the proposal that exploitation is the predominant function of the ventral visual pathway in primates.

Conversely, by the same logic, the lack of a tight visual topography in areas of the dorsal stream, large receptive fields, and extensive input projections from the visual periphery with low spatial resolution, together imply weak signal for feature preference and less (but not absent) selectivity for visual features in the responses of cells located in areas of the dorsal stream. Instead, cells in areas of the dorsal stream typically respond vigorously to flashing or moving spots of light (Figure 6). The response properties of the cells in the dorsal stream are in accord with the theme of exploration: scan the space for salient stimuli in order to judge where next to deploy neural resources, with some regard for featural properties of the stimuli.

This does not mean that the dorsal stream has no role at all in exploitation, e.g., the dorsal stream contributes, as mentioned above, to object recognition in some ways, i.e., when the stimuli are novel, unconventional or challenging in some way (stimuli for which the ventral visual pathway does not have pre-existing representations and cooperation across pathways and areas is necessary), when the integration of features across a large area of space, or across multiple fixations, is required (for which the large receptive fields of cells in the dorsal stream that cover the center as well as the periphery are better-suited and in which stable representations over multiple fixations can emerge), or when novel stimulus-response associations or perceptual classifications are to be learnt *de novo* (for which the ventral pathway does not have the flexibility in learning new perceptual classifications based on hard-wired visual features richly represented in the ventral visual pathway). In short, the dorsal stream has a primary role in the exploration of space and a role in the exploitation of visual information. The dual role of the dorsal stream and redundancy of function that the dorsal stream provides is in accord with the fact that the dorsal stream represents the visual center along with the ventral stream, thus providing redundancy in the brain's representation of (central) visual space, rendering it suitable for serving dual roles.

## Conclusion

To sum up, we argue for a more complete, encompassing segregation into dorsal and ventral visual pathways that stems from an anatomical segregation of complete or global, and central vision that begins at the level of the retina and continues downstream into striate cortex and into higher-order cortex. The proposal of segregating on the basis of central vs. overall vision hews closely to the theme of sensory underpinning rather than function. Function arises as an offshoot or consequence of sensory underpinning^12^. The ventral visual pathway of primates, because it is focused on the processing of central vision, is involved in the exploitation of visual information, whereas the dorsal pathway, because it processes information from a broader, more complete expanse of space, is involved in the exploration of the environment. Because the dorsal pathway's coverage of visual space subsumes the ventral visual pathway's coverage of visual space, the dorsal pathway is involved in exploitation of the environment in certain conditions as described above, as well. Our scheme can thus be construed as Müller (1837) labeled line law of specific nerve energies but recast in modern context.

The model, grounded in the proposal that the segregation depends on the dichotomy between focal vision (limited, expensive resources devoted to a small portion of visual space) and global vision (expansive resources devoted to coarsely monitoring the entire environment), builds on historical antecedents: Trevarthen (1968) proposed a model for vision in monkeys in which he proposed two streams, one each responsible for ambient and focal vision; Norman (2002) furnished a list of differences between the two processes of his dual process model and one of the differences stated was the source of the visual input: foveal/parafoveal vs. from all across the retina; however, no accounting was provided of how these differences coalesced or if and how any of the putative differences could be the principal driver. Our exploitation/exploration framework goes beyond these historical antecedents in several crucial ways. The exploitation/exploration dichotomy is novel; it provides a unifying framework for previous models of dual stream processing, accounts for diverse functions proposed at various times for each of the two streams, and provides a more complete and reasoned basis for diverse experimental findings heretofore unaccounted for by prior models.

## Author contributions

BRS formulated the hypothesis and BRS and RY jointly reviewed the literature for detailing the model. BRS and RY wrote the paper.

### Conflict of interest statement

The authors declare that the research was conducted in the absence of any commercial or financial relationships that could be construed as a potential conflict of interest.

## Footnotes

^1^Owing to the fact that these studies were conducted on anesthetized monkeys, it cannot be tested if activation of these areas actually contributed toward shape and/or object perception.

^2^Others may report slightly different proportions; however, we note that our estimates are based on studies that purportedly focused on measuring receptive field properties of neurons and contend that our estimated proportions are reasonable approximation to the actual values.

^3^Our proposal is premised on the idea that ambient coverage of visual space is suited for exploration, and limited, focused coverage of space is more suited for exploitation. We argue that a similar principle applies to receptive field dynamics in individual brain areas, i.e., the dynamic alterations in receptive field location and size that occur from eye movements to a target can be interpreted using the exploitation/exploration theme. In over two-thirds of the neurons recorded in LIP under fixation versus free gaze conditions (Ben Hamed et al., 2002), “…proportionally larger amount of neuronal resources were devoted to central space during fixation, and an attenuated center to periphery gradient in the visual field representation during free gaze” was observed—in other words, sharper focus on the visual center during exploitation and diffuse, more uniform representation of visual space during exploration (It bears mention that shifts in the peak of the receptive field were modest—slightly larger than 1° on average; however, the center of mass of RF did not vary significantly between the two conditions (< 0.2°), indicating a redistribution of activity within the RF rather than a full-scale change in RF location. That is to say, the dynamic shifts are small). Similar findings, viz. receptive fields shrink and shift toward the saccadic target, were observed in area V4 (Tolias et al., 2001).

^4^The segregation of the brain into a stream favoring focal processing at a high resolution and another stream favoring more global processing—albeit coarsely—could be applicable to sensory modalities besides vision and to species other than primates, but only under certain specific conditions. The primary constraint for such development is brain mass/size, as mentioned at article's outset. Species with smaller brains are more likely to (afford to) have all their brain areas connect to one another, with the result being that there is less pressure for the segregation and development of parallel processing streams (Striedter, 2005). Species that have large brains and a number of brain areas have high wiring costs and have to economize connectivity and are therefore more likely to show a development of parallel processing streams (Striedter, 2005). We are not aware of species other than primates that fulfill these constraints in full. The mustache bat is relatively small in size (Neuweiler, 2000), its brain is somewhat large; it has an acoustic fovea in auditory cortex (areas DSCF) and an overrepresentation of certain auditory frequencies. There are some signs pointing to the presence of three processing pathways in auditory cortex (Fitzpatrick et al., 1998): the first pathway involving the DSCF, cortical areas AV and (parts of) DIF, and the other two pathways involving other areas of auditory cortex (second pathway: CF/CF, a different part of DIF, and area VA; third pathway involving areas VF, DF and FM/FM). The function of the first pathway involving the acoustic fovea is to acquire high quality, high temporal resolution information about the acoustic scene, specifically the detection of rapid Doppler fluctuations, i.e., wing flutter (Ulanovsky and Moss, 2008); this is reminiscent of the functions we have proposed for the analogous pathway in primates.

^5^Even within area in Gattass et al. (1981), natively directionally untuned neurons in LIP showed more flexibility than natively tuned neurons: LIP neurons that were not direction selective during passive viewing showed larger directional tuning shifts during the delayed conjunction matching task than ones that were. These results are consistent with our proposal.

^6^It bears mention that the modulation of V4 neurons is also greater when color was the matching criterion; however, a higher percentage of V4 cells showed significant cue effects for direction matching than for color matching, in accord with our argument.

^7^The discrimination required was rather coarse: the stimuli used in the study were rather large—9° in diameter in their first experiment, 5.6° in their second experiment, and 10° in the third and final experiment). The stimuli used were made up of connected line segments forming random angles, i.e., were relatively novel. The dorsal pathway is well-suited for form discrimination over large areas of visual space, involving integration over multiple retinal images over time and space, especially of novel stimuli heretofore not seen in everyday life.

^8^Saliency can be biological (i.e., innate, driven by rewards of food, drink, mating etc.) or behavioral (associated, via training or experience, with said rewards or goals, e.g., in a laboratory task in which experimenter trains a macaque to point to/reach/gaze at red, vertical items in the periphery over red, horizontal ones and green, vertical ones).

^9^Audiovisual convergence and integration are not ubiquitous in the ventral pathway either. The superior temporal sulcus (STS), a multisensory area that does not belong to either the dorsal or ventral stream (and its macaque homolog—STP), has been consistently associated with audiovisual integration (Calvert et al., 2000; Beauchamp, 2005), and speech recognition, in particular (Raij et al., 2000; Sekiyama et al., 2003; Saito et al., 2005). Neuroimaging studies of various audiovisual effects, e.g., the double flash illusion, the ventriloquist effect, and the McGurk effect again show neural basis in areas that are not typically associated with the ventral pathway. Areas V1, STS, and the superior colliculus are differentially activated, under conditions of identical physical stimulation, when the double-flash illusion is perceived versus when it is not. Studies of the ventriloquist effect combining fMRI and EEG (Bonath et al., 2007) have found activation of the planum temporale (a cortical area just posterior to the auditory cortex within the sylvian fissure) only on trials where the ventriloquism illusion occurred; a second fMRI study found alteration of the neural pattern in the BOLD signals in the posterior superior temporal gyrus or pSTG, mainly in the right pSTG (Callan et al., 2015): Areas of the ventral stream do not appear to be involved in the ventroliquist effect. fMRI studies of the McGurk effect (Skipper et al., 2007) also found percept related activity (rather than activity corresponding to the actual physical pattern) initially in the ventral premotor cortex, which is a frontal area related to speech production, and only stimulus-evoked activity in the visual and other sensory areas, which was later replaced by percept related activity—again, no clear role for areas of the ventral stream was observed in the McGurk audiovisual percept; an MEG study of the McGurk effect claimed that the distribution of MEG activity corresponding to the percept was best explained by dipoles, i.e. sources, in the auditory cortex and surrounding belt areas (Sams et al., 1991)—again, no areas of the ventral stream were implicated.

^10^Single-unit recordings in area VIP in the primate have found that across the population recorded, neuronal response is uniform across FOE location (Bremmer et al., 2002). Stimuli used in the study were 70 × 70°full-field optic flow patterns; the individual contributions of the foveal and peripheral optic flow signals (by blanking corresponding portions of the visual field) to neuronal response (in particular in an ecological setting, i.e., when the FOE is located at the fovea) were not explored.

^11^Analogous to our proposal on the suitability of the ventral stream for object recognition and feature vision are recent experimental data suggesting eccentricity bias as an organizing principle for object recognition within higher-order cortex (Levy et al., 2001; Hasson et al., 2002). The recognition of objects requiring high resolution, e.g., faces, letter strings, and words, activates center-biased representations in cortex (e.g., faces activate posterior fusiform gyrus), whereas objects requiring low resolution, e.g., buildings and tools activates peripheral representations as well (e.g., buildings activate regions along the collateral sulcus/parahippocampal gyrus).

^12^It is plausible that in evolution, the parts of the retina that were close to the center of gaze were anatomically segregated from the parts of the retina that were away from it. Then, when acuity zones started forming in the retina, i.e., the development of a fovea, across evolution, it simply piggybacked on this already existing anatomical segregation.
